# Supplementary material for: Cytoplasmic accumulation of a splice variant of hnRNPA2/B1 contributes to FUS-associated toxicity in a mouse model of ALS
Source: Cell Death Dis. 2025 Mar 29;16(1):219. doi: 10.1038/s41419-025-07538-8 (PMC11954880; doi:10.1038/s41419-025-07538-8)

Figure 1C

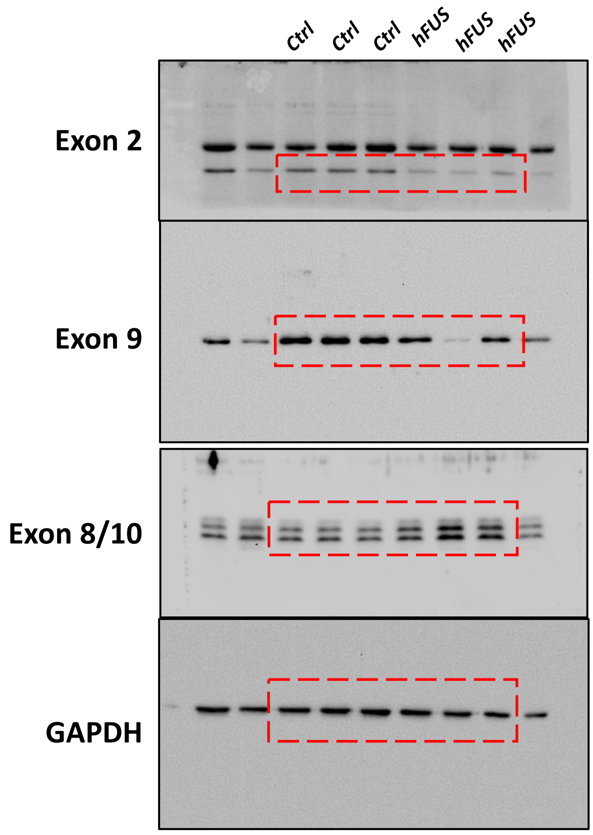

Figure 1D

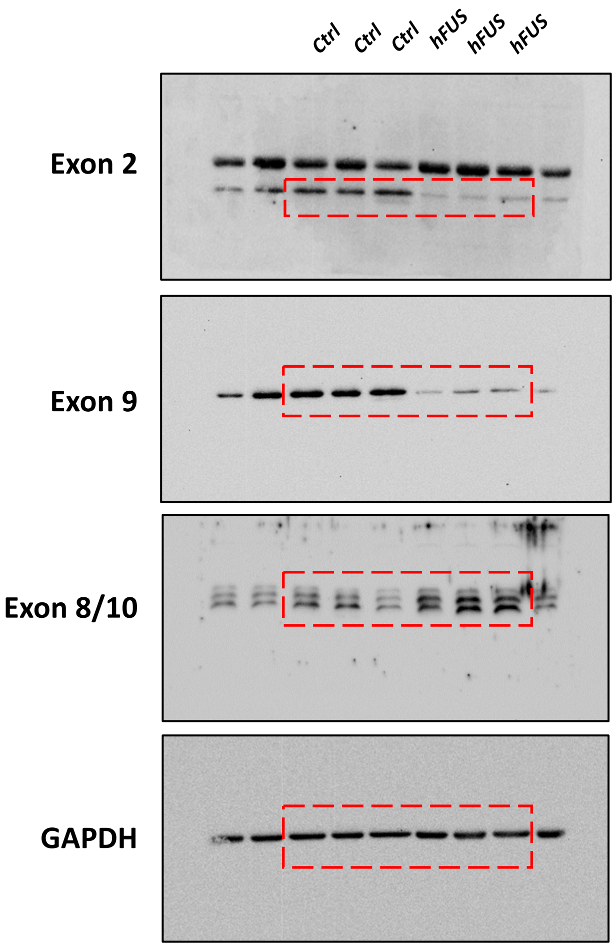

Figure 3C

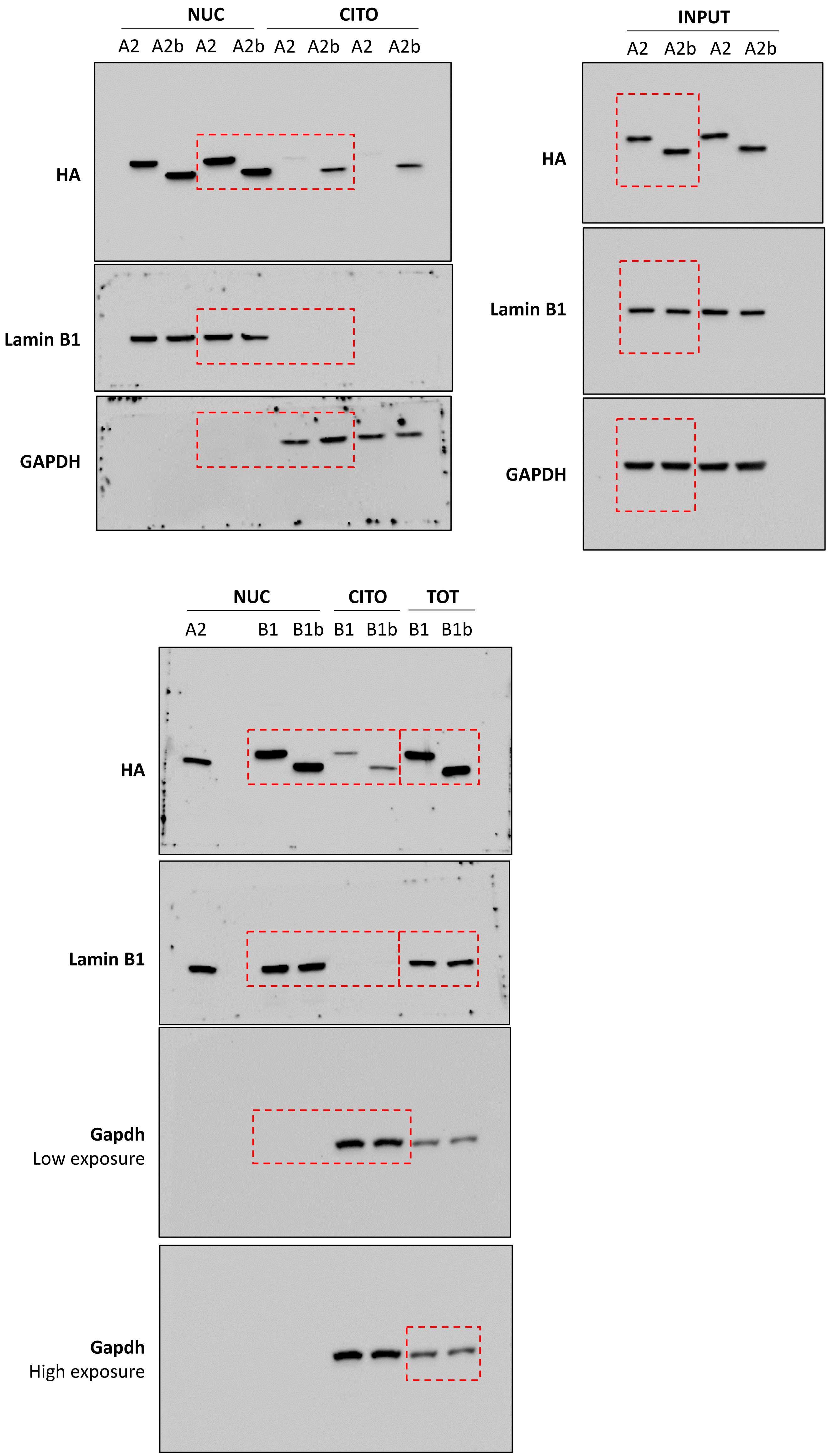
NUC
CITO
A2 A2b A2 A2b A2 A2b A2 A2b
HA
Lamin B1
GAPDH
INPUT
A2 A2b A2 A2b
HA
Lamin B1
GAPDH
NUC
CITO
TOT
A2 B1 B1b B1 B1b B1 B1b
HA
Lamin B1
Gapdh
Low exposure
Gapdh
High exposure

Figure 4C

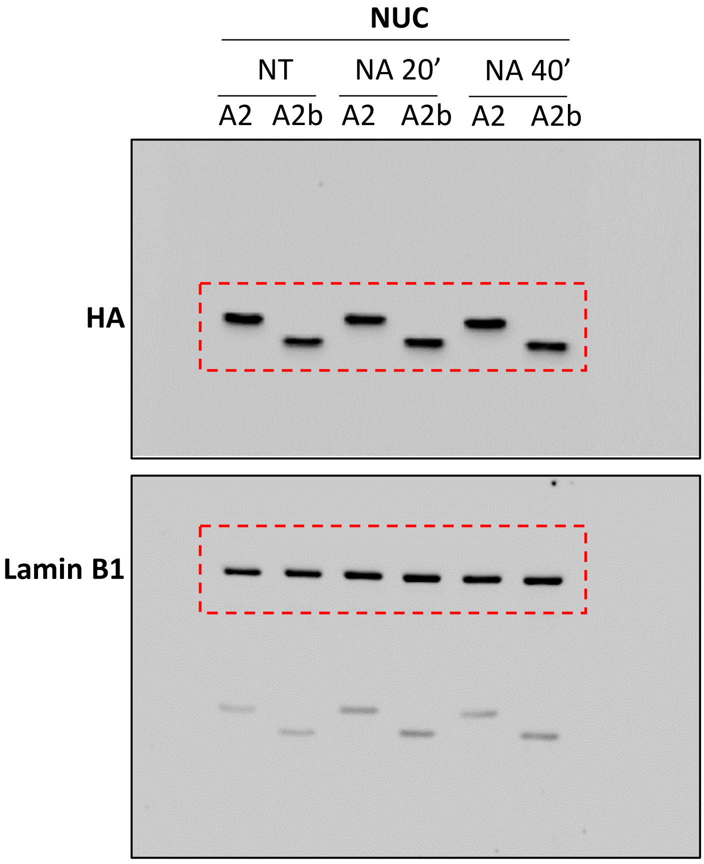
NUC
NT
NA 20'
NA 40'
A2 A2b A2 A2b A2 A2b
HA
Lamin B1

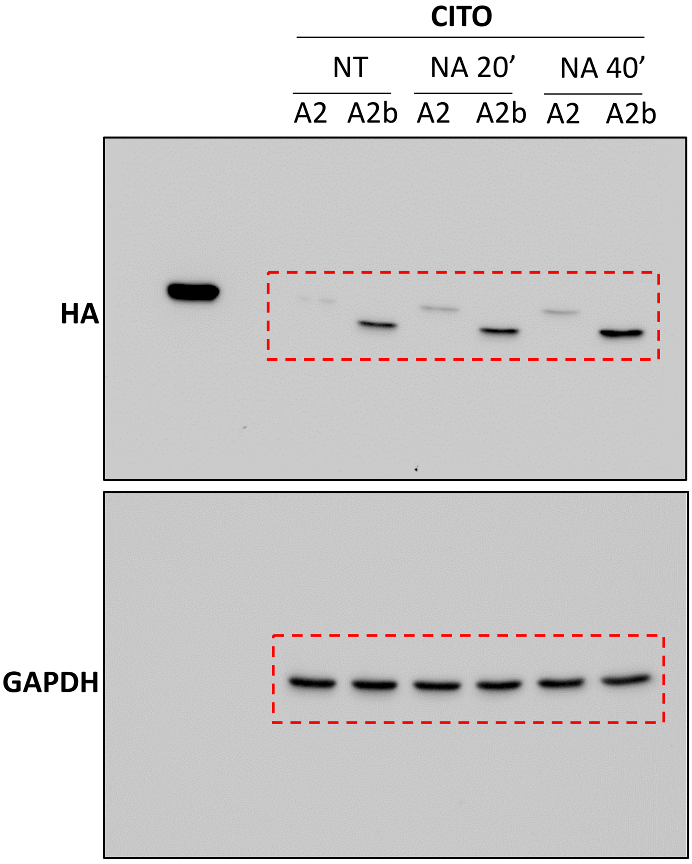
CITO
NT
NA 20'
NA 40'
A2 A2b A2 A2b A2 A2b
HA
GAPDH

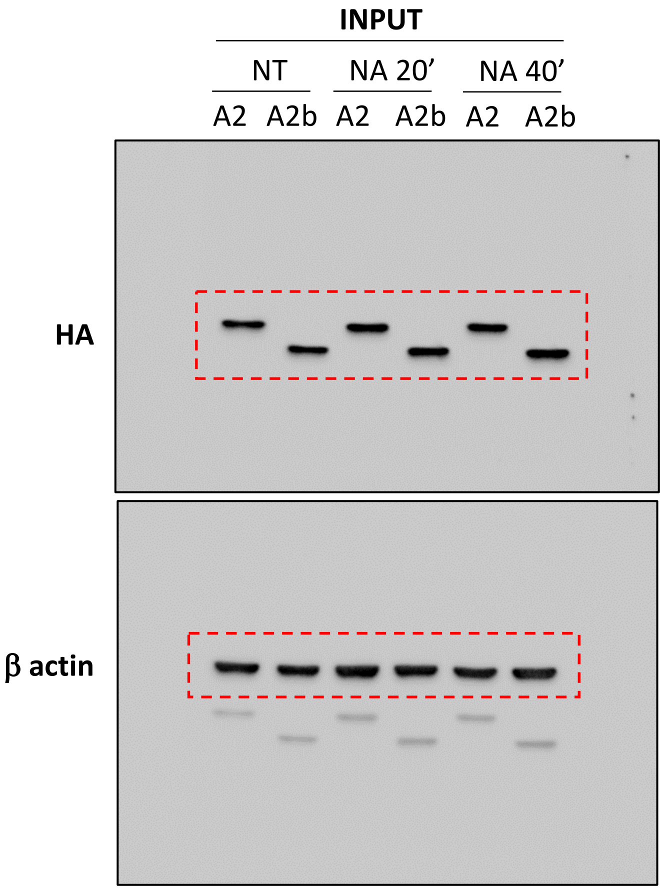
INPUT
NT
NA 20'
NA 40'
A2 A2b A2 A2b A2 A2b
HA
β actin

Figure 5A

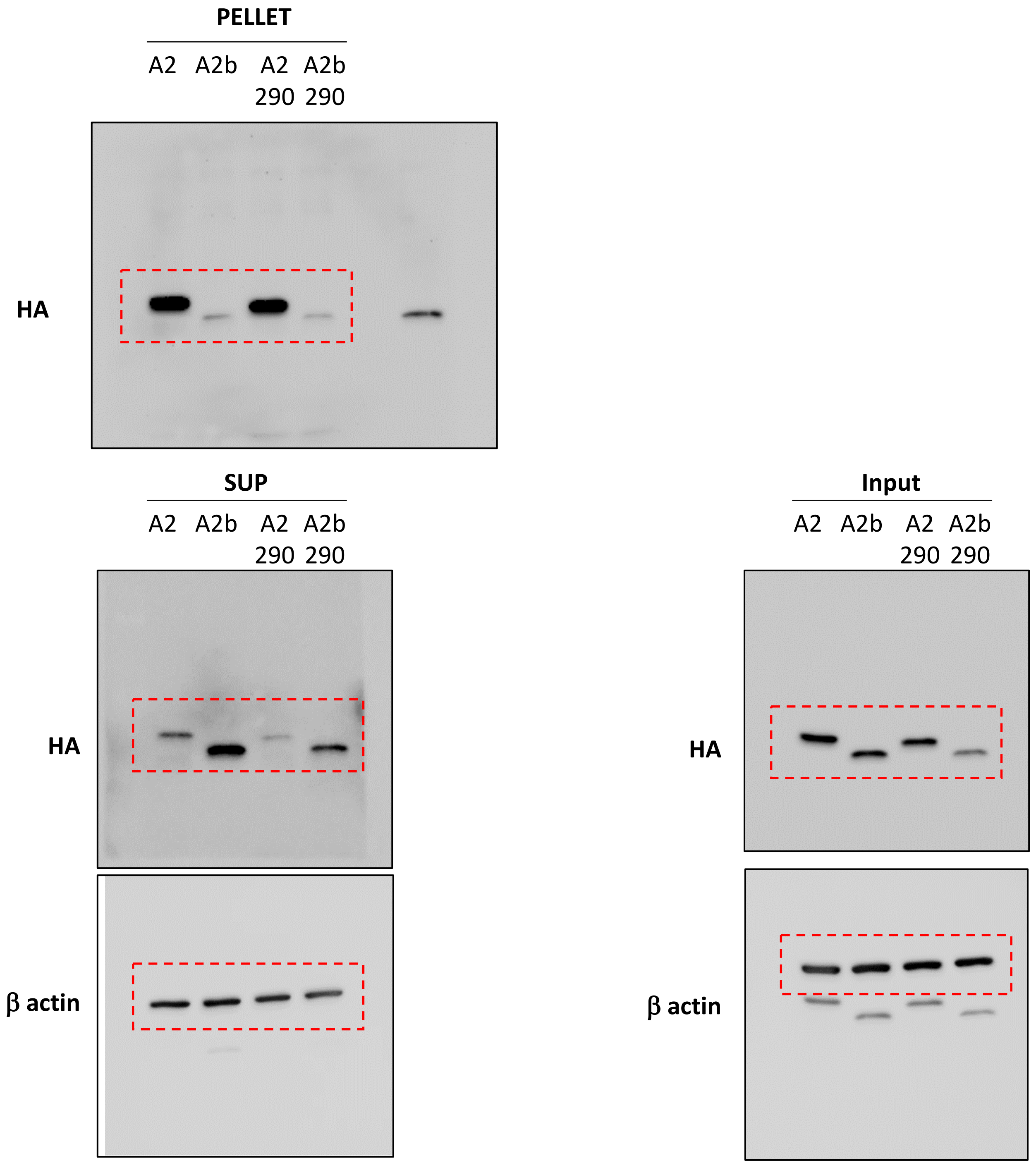
PELLET
A2
A2b
A2 290
A2b 290
HA
SUP
A2
A2b
A2 290
A2b 290
HA
β actin
Input
A2
A2b
A2 290
A2b 290
HA
β actin

Figure 6D

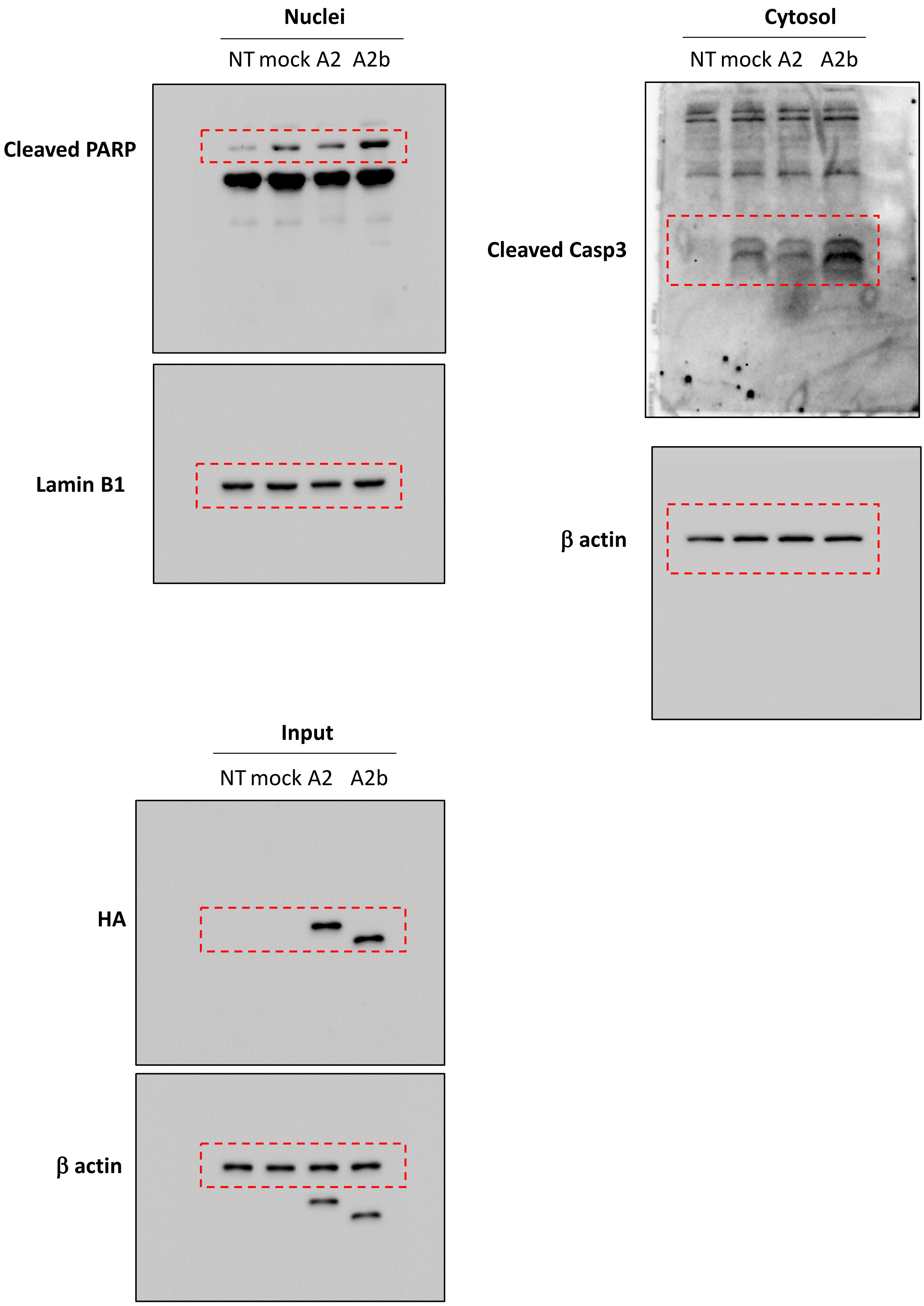

Nuclei
NT mock A2 A2b
Cleaved PARP
Lamin B1
Cytosol
NT mock A2 A2b
Cleaved Casp3
β actin
Input
NT mock A2 A2b
HA
β actin

Figure 6E

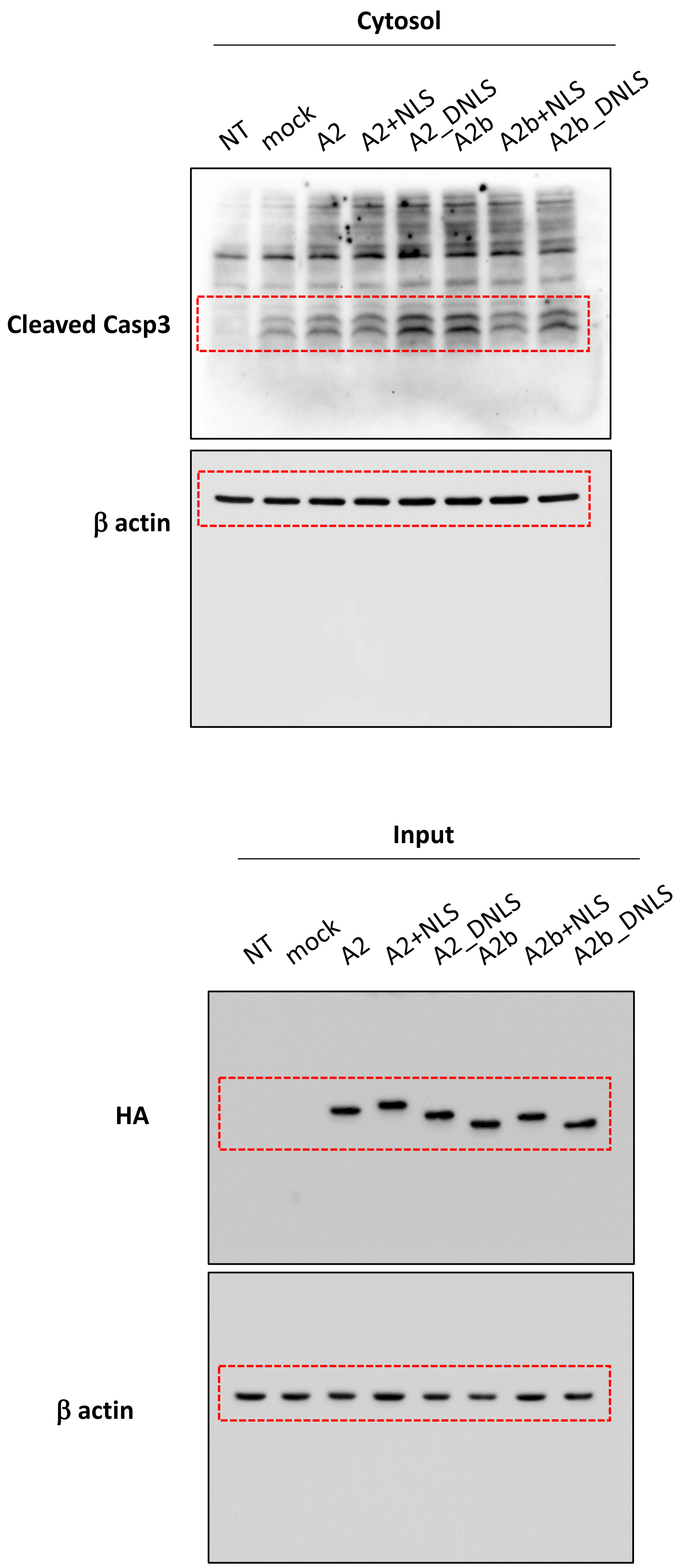
Cytosol
NT
mock
A2
A2+NLS
A2_DNLS
A2b
A2b+NLS
A2b_DNLS
Cleaved Casp3
β actin
Input
NT
mock
A2
A2+NLS
A2_DNLS
A2b
A2b+NLS
A2b_DNLS
HA
β actin

Supplementary Figure 1

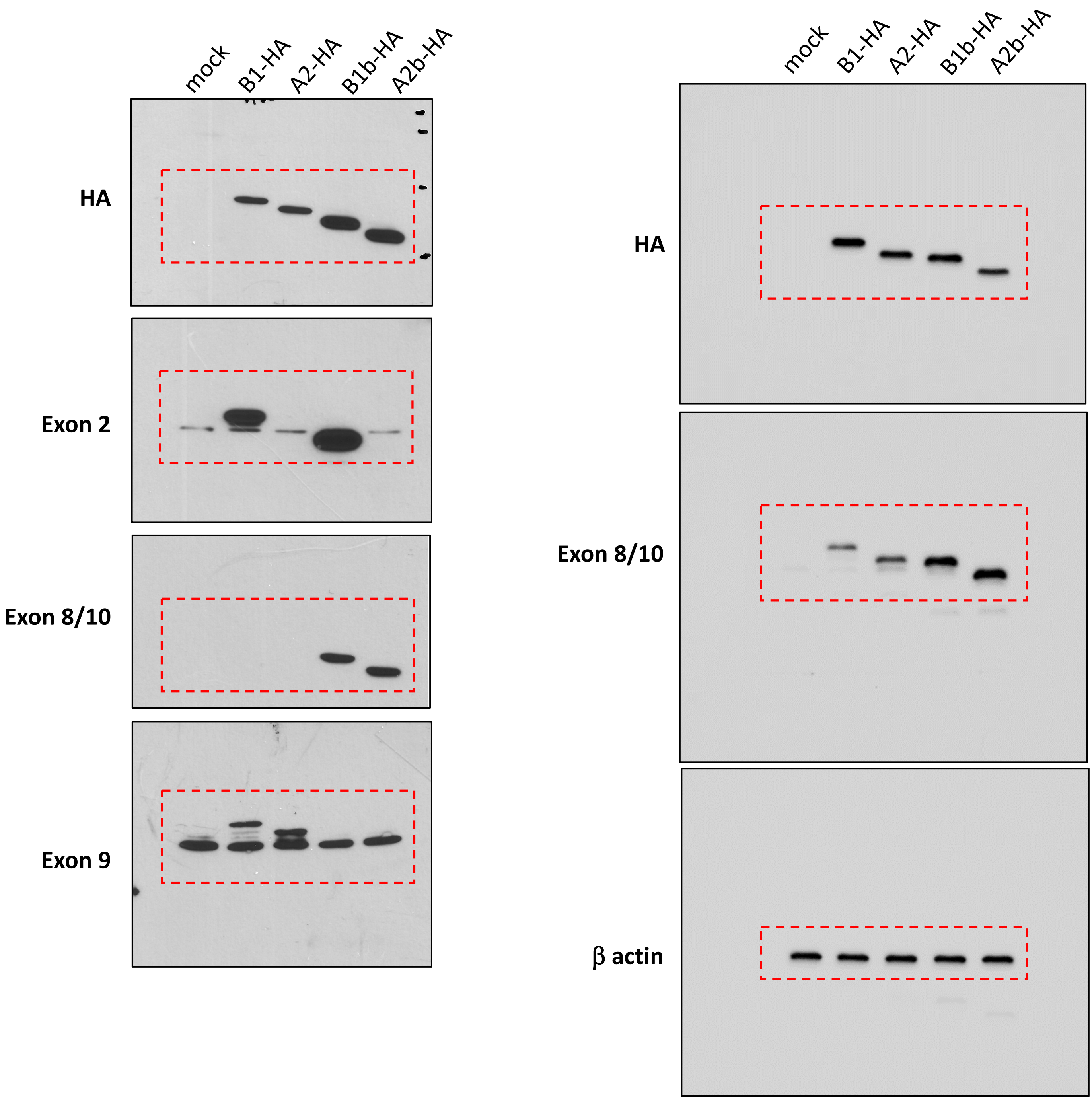

Supplementary Figure 7B

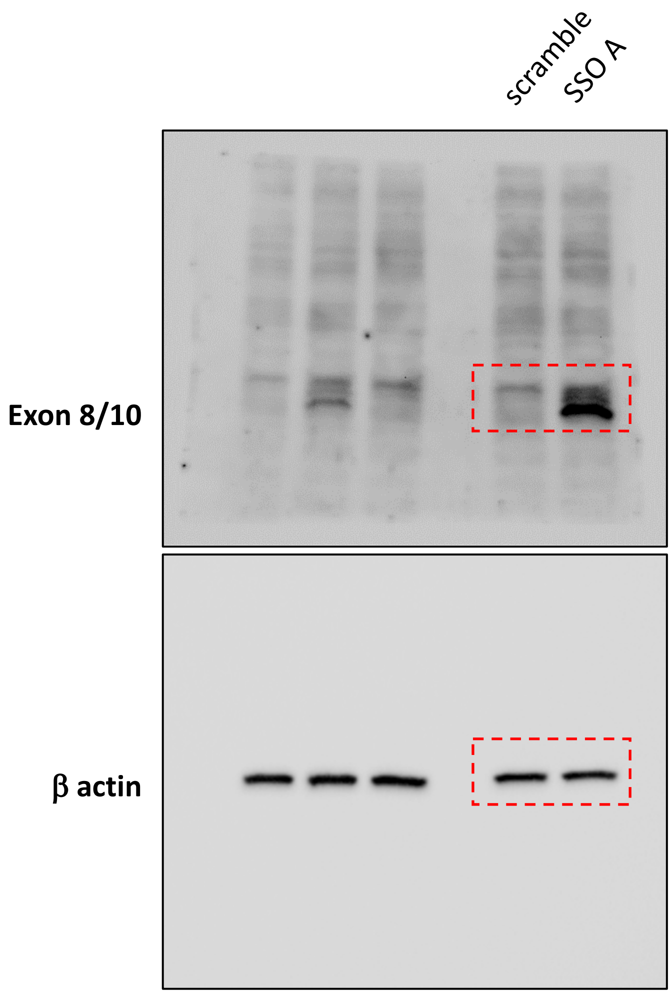

Supplementary Figure 8E

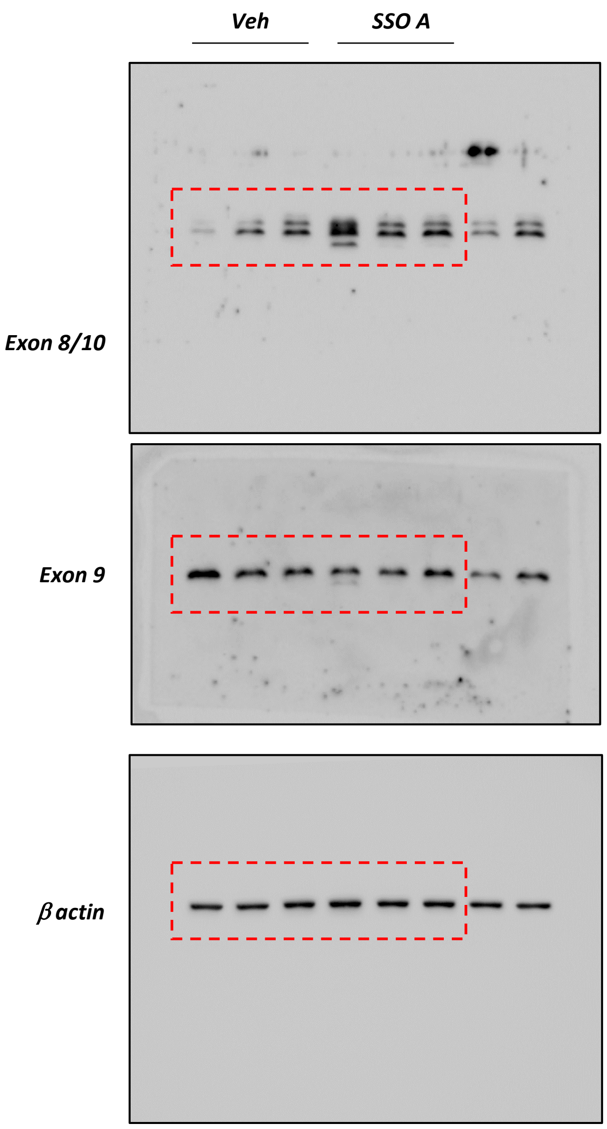

Supplement: Supplementary file 2 — SUPPLEMENTARY MATERIAL-ORIGINAL WESTERN BLOT [file 41419_2025_7538_MOESM2_ESM.pdf]
